# Comparison of Biliary Complications Rates After Brain Death, Donation After Circulatory Death, and Living-Donor Liver Transplantation: A Single-Center Cohort Study

**DOI:** 10.3389/ti.2022.10855

**Published:** 2022-12-09

**Authors:** Raphael Pascal Henri Meier, Yvonne Kelly, Hillary Braun, Daniel Maluf, Chris Freise, Nancy Ascher, John Roberts, Garrett Roll

**Affiliations:** ^1^ University of California, San Francisco, San Francisco, CA, United States; ^2^ University of Maryland, Baltimore, Baltimore, MD, United States

**Keywords:** liver transplantation, living donors, donation after brain death, donation after circulatory death, biliary anastomotic stricture, ischemic cholangiopathy, bile leak

## Abstract

Donation-after-circulatory-death (DCD), donation-after-brain-death (DBD), and living-donation (LD) are the three possible options for liver transplantation (LT), each with unique benefits and complication rates. We aimed to compare DCD-, DBD-, and LD-LT-specific graft survival and biliary complications (BC). We collected data on 138 DCD-, 3,027 DBD- and 318 LD-LTs adult recipients from a single center and analyzed patient/graft survival. BC (leak and anastomotic/non-anastomotic stricture (AS/NAS)) were analyzed in a subset of 414 patients. One-/five-year graft survival were 88.6%/70.0% for DCD-LT, 92.6%/79.9% for DBD-LT, and, 91.7%/82.9% for LD-LT. DCD-LTs had a 1.7-/1.3-fold adjusted risk of losing their graft compared to DBD-LT and LD-LT, respectively (*p* < 0.010/0.403). Bile leaks were present in 10.1% (DCD-LTs), 7.2% (DBD-LTs), and 36.2% (LD-LTs) (ORs, DBD/LD vs. DCD: 0.7/4.2, *p* = 0.402/<0.001). AS developed in 28.3% DCD-LTs, 18.1% DBD-LTs, and 43.5% LD-LTs (ORs, DBD/LD vs. DCD: 0.5/1.8, *p* = 0.018/0.006). NAS was present in 15.2% DCD-LTs, 1.4% DBDs-LT, and 4.3% LD-LTs (ORs, DBD/LD vs. DCD: 0.1/0.3, *p* = 0.001/0.005). LTs w/o BC had better liver graft survival compared to any other groups with BC. DCD-LT and LD-LT had excellent graft survival despite significantly higher BC rates compared to DBD-LT. DCD-LT represents a valid alternative whose importance should increase further with machine/perfusion systems.

## Graphical Abstract

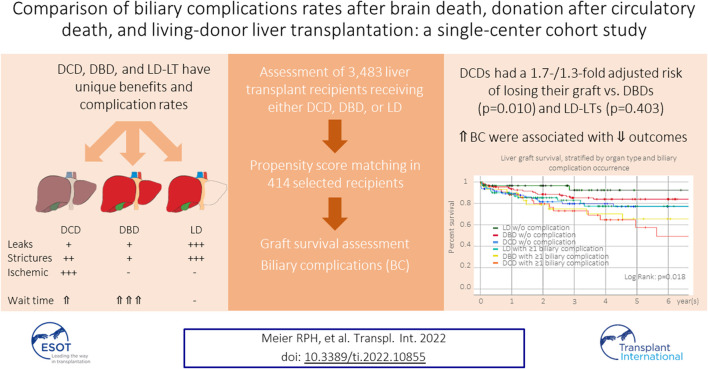

## Introduction

In regions with a high average Model for End-stage Liver Disease (MELD) score at transplant, organs from donation after brain death (DBD) donors often go to the sicker patients with high MELD scores, and so for patients with liver cancer and/or a low MELD score, organs from donation-after-circulatory-death (DCD) donors and living donors (LD) [1, 2] represent alternatives for liver transplantation (LT). DCD donors are increasingly used for LT in an effort to address organ scarcity and to decrease waiting-list mortality [3]. It is well recognized that DCD livers expose the recipient to increased risk from the inevitably longer donor warm ischemia time (dWIT). Aside from primary nonfunction [4], the most feared complication, and one of the main reasons for graft loss, is ischemic cholangiopathy (IC), defined as the appearance of intrahepatic non-anastomotic biliary strictures (NAS), which occurs in 10%–50% of cases [5–9]. The increasing use of normothermic preservation machines (NMP) might significantly modify these complication rates [10]. However, to date, NMP is not broadly available, and many US centers still avoid DCDs or apply very strict donor selection criteria [9]. In this regard, we and others have developed scores to select donors/recipients in order to optimize outcomes with a special emphasis on minimizing biliary complications [11–14]. Known risk factors for IC are donor age (>40 years) [6, 15], prolonged cold ischemic time (CIT) (>8 h) [6], prolonged dWIT (>20 min), low venous oxygen saturation (SvO_2_ ≤ 60) [15], and donor liver extraction time [8, 13]. Besides IC, other relevant ischemic complications include anastomotic biliary strictures (AS) and bile leaks which were previously shown to range between 10% and 15% in DCD cases, and not be significantly different from DBD rates [6]. Just as the use of DCD grafts has increased in recent years, so has the use of LD-LT in order to further increase organ availability [16]. The outcomes are overall excellent [17], however, a higher risk of biliary complication is present as well with anastomotic biliary stenosis and leak ranging from 10% to 35% in different series [16, 18–21]. The difficulties encountered by patients experiencing recurrent biliary issues added to the minimal, but a non-null, risk to the living donor [22] and variable access to LD, warrants a thorough assessment and selection of both donor and recipient by the transplant team.

For a given patient with all three options, the choice might be difficult to make since each modality has unique benefits, risks, and potential complications. We sought to compare biliary complications and graft survival between DCD-, DBD-, and LD-LT at a single center, with the intention to provide more data for guiding the decision between these three possible options for transplantation.

## Methods

### Study Design and Patients

Approval was obtained by the Institutional Review Board of the University. Donor and recipient data were extracted from the UNOS database and included all consecutive adult liver transplants performed at the University Medical Center between 1989 and 2019 (*n* = 3,483), which included 138 DCD, 318 LD, and 3,027 DBD (Table 1). 138 DCD-LTs were compared to 138 DBD-LTs (selected using a propensity score matching technique), and 138 randomly selected LD-LTs. Ischemia times were defined as previously described [13]. Donor and recipient selection and procedures were performed as previously described [13, 23, 24]. DCD grafts were procured using the super-rapid technique with local modifications [25]. Ischemic cholangiopathy was defined by the presence of intrahepatic, non-anastomotic biliary strictures (NAS) and dilatations occurring in the absence of ductopenic rejection or recurrent primary sclerosing cholangitis. When suspected (increased alkaline phosphatase and bilirubin), NAS was diagnosed on endoscopic retrograde cholangiopancreatography (ERCP) and/or Magnetic Resonance Imaging (MRI). One DBD recipient developed secondary NAS after a hepatic artery thrombosis. The occurrence of AS and biliary leaks were collected from patients’ chart reviews. The median follow-up was 6 years (min-max, 0–29 years) for the entire cohort (*n* = 3,483) and 3 years (min-max, 0–27 years) for the 1:1 control cohort (*n* = 414). In the entire cohort (*n* = 3,483), MELD had 1% missing data, recipient BMI and CIT had 6% missing data, and all the other variables had no missing data. In the 1:1 matched control cohort (*n* = 414), CIT and dWIT had 1% missing data, and all the other variables had no missing data.

**TABLE 1:** Recipient and donor baseline characteristics of donation after cardiac death, donation after brainstem death, and living donor liver transplantation.

Characteristics	DCD LT (*n* = 138)	DBD LT (*n* = 3,027)	LD LT (*n* = 318)	P-value^a^	P-value^b^
Recipient					
Age at transplant, years	57.5 ± 9.0	53.3 ± 10.7	53.9 ± 11.1	<0.001	<0.001
Gender (%)					
Male	103 (74.6)	1,942 (64.2)	158 (49.7)	0.012	<0.001
Female	35 (25.4)	1,085 (35.8)	160 (50.3)		
Pretransplant BMI, kg/m^2^	27.9 ± 5.9	27.3 ± 5.9	26.2 ± 4.6	0.236	0.003
Ethnicity (%)					
American Indian	2 (1.4)	33 (1.1)	2 (0.6)	0.428	0.652
Asian	18 (13.0)	491 (16.2)	27 (8.5)		
Black	4 (2.9)	180 (5.9)	11 (3.5)		
Native Hawaiian	1 (0.7)	30 (1.0)	2 (0.6)		
Hispanic	38 (27.5)	658 (21.7)	81 (25.5)		
Multiracial	0 (0.0)	16 (0.5)	1 (0.3)		
White	75 (54.3)	1,619 (53.5)	194 (61.0)		
Etiology					
A1AT	1 (0.7)	13 (0.4)	2 (0.6)	<0.001	<0.001
Auto-immune	4 (2.9)	81 (2.7)	13 (4.1)		
Amyloidosis	1 (0.7)	0 (0.0)	0 (0.0)		
Biliary atresia	1 (0.7)	2 (0.1)	3 (0.9)		
Cholangiocarcinoma	2 (1.4)	4 (0.1)	0 (0.0)		
Cryptogenic	3 (2.2)	205 (6.8)	23 (7.2)		
EtOH	29 (21.0)	363 (12.0)	41 (12.9)		
HBV	12 (8.7)	324 (10.7)	24 (7.5)		
HCV	68 (49.3)	874 (28.9)	84 (26.4)		
NASH	9 (6.5)	132 (4.4)	25 (7.9)		
Other	0 (0.0)	780 (25.8)	39 (12.3)		
PBC	2 (1.4)	112 (3.7)	28 (8.8)		
PSC	3 (2.2)	121 (4.0)	36 (11.3)		
Wilson	3 (2.2)	16 (0.5)	0 (0.0)		
HCC					
Presence	40 (29.0)	586 (19.4)	50 (15.7)	0.005	0.001
Absence	98 (71.0)	2,441 (80.6)	268 (84.3)		
Median MELD, IRQ	23 (12–32)	38 (31–40)	18 (13–26)	<0.001	0.008
Era					
1989–2000	0 (0.0)	912 (30.1)	10 (3.1)	<0.001	<0.001
2001–2010	26 (18.8)	1,038 (34.3)	128 (40.3)		
2011–2018	112 (81.2)	1,077 (35.6)	180 (56.6)		
Donor factors					
Age, years	31.7 ± 10.3	39.5 ± 16.7	36.5 ± 10.8	<0.001	<0.001
Gender (%)					
Male	92 (66.7)	1,803 (59.6)	163 (51.3)	0.096	0.002
Female	46 (33.3)	1,224 (40.4)	155 (48.7)		
BMI, kg/m^2^	25.5 ± 5.2	26.5 ± 6.1	25.8 ± 4.4	0.041	0.557
Ethnicity (%)					
American Indian	0 (0.0)	19 (0.6)	0 (0.0)	0.132	0.070
Asian	4 (2.9)	226 (7.5)	28 (8.8)		
Black	11 (8.0)	234 (7.7)	11 (3.5)		
Hispanic	34 (24.6)	677 (22.4)	69 (21.7)		
Multiracial	4 (2.9)	30 (1.0)	6 (1.9)		
Native Hawaiian	0 (0.0)	27 (0.9)	1 (0.3)		
Unknown	0 (0.0)	9 (0.3)	0 (0.0)		
White	85 (61.6)	1,805 (59.6)	203 (63.8)		
Cause of death					
Anoxia	73 (52.9)	579 (19.1)	NA	<0.001	NA
Cerebrovascular	17 (12.3)	1,213 (40.1)			
CNS tumor	0 (0.0)	8 (0.3)			
Head trauma	41 (29.7)	1,061 (35.1)			
Not reported	0 (0.0)	9 (0.3)			
Other	7 (5.1)	157 (5.2)			
Cold ischemic time, hours	7.7 ± 2.6	9.0 ± 3.9	2.4 ± 2.6	<0.001	<0.001
Donor warm ischemia time, minutes	20 ± 6	NA	NA	NA	NA
Donor hepatectomy time, minutes	41 ± 16	NA	NA	NA	NA

Data are presented as mean ± standard deviation or n (%), unless specified otherwise.

DCD, donation after cardiac death; DBD, donation after brainstem death; LD, living donor; LT, liver transplantation; BMI, body mass index, EtOH, ethanol use; HBV, hepatitis B virus; HCV, hepatitis C virus; NASH, nonalcoholic steatohepatitis; PBC, primary biliary cholangitis; PSC, primary sclerosing cholangitis; A1AT, alpha-1 antitrypsin; MELD, Model For End-Stage Liver Disease; CNS, central nervous system; IRQ, interquartile range.

^a^ DCD versus DBD.

^b^ DCD versus LD. Student t-test for continuous variables, X^2^ test for binary or categorical variables (global *p*-value).

### Patient Selection, Organ Allocation, and Operation

Patients diagnosed with end-stage liver disease were evaluated for candidacy by a multidisciplinary team and placed on the transplant waiting list [24]. Before 2002, the United Network for Organ Sharing (UNOS) criteria were used to determine priority (no DCD-LT was performed during this time). From 2002 to present, the MELD allocation system has been used [26]. Organ selection and LT were performed as previously described [24]. All liver grafts were perfused with University of Wisconsin solution (hepatic artery and portal vein). LT was performed as previously described [24], typically utilizing the piggyback technique and duct-duct biliary anastomosis.

### Statistical Analysis

Continuous variables were expressed as means, and standard deviations (SD) and categorical variables were expressed as counts and percentages. Comparison between groups was performed using the Student’s t-test for continuous variables and the chi-squared test for binary or categorical variables. Propensity score matching for each patient was generated using a multivariable binary logistic regression model. DCD patients were matched 1:1 with DBD patients using recipient age, sex, and pretransplant BMI as well as donor age, sex, BMI, and cold ischemia time as a covariate with a caliper of 0.01. Due to the lower number of LD cases and the limited value of selecting one specific matching variable over another, 138 LD-LT recipients were randomly selected for comparison. To ensure that random matching was appropriate, we performed a sensitivity analysis using optimal full propensity score matching restricted to observations that had propensity scores in the extended common support region (0.06–0.75). Acceptable balance was defined by a maximum of 0.1 for the absolute value of standardized difference and by values within the 0.5–2 range for variance ratio. Survival analyses were performed using the Kaplan–Meier method and the log-rank test. Uni-/multivariate Cox proportional-hazard regression was used to compute hazard ratios (HR). We used IBM SPSS Statistics version 26 and SAS version 9.4 for all computations (IBM Corp. Armonk, NY). Ninety-five percent confidence intervals (95%CI) were reported, and an exact two-sided *p*-value <0.05 was considered statistically significant.

## Results

### Patient Characteristics

**FIGURE 1:**
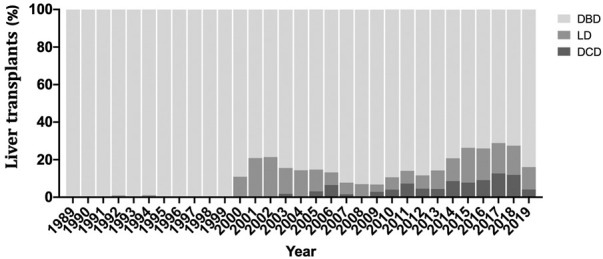
Percentage of recipient receiving a donation after cardiac death (DCD), donation after brainstem death (DBD), or living donor liver transplantation (LD) over the study period.

During the study period, 3,483 liver transplants were performed, including 138 DCD, 3,027 DBD and 318 LD (Figure 1; Table 1). Compared to DBD, DCD recipients were significantly older and more likely to be males. The top two indications in DCD-LTs were cirrhosis from alcohol (EtOH) use and hepatitis C virus (HCV), and more recipients had hepatocellular carcinoma (HCC) compared to DBD-LTs. The median (interquartile range (IRQ)) MELD in DCD recipients was 23 (12–32) versus 38 (31–40) in DBD recipients (*p* < 0.001). DCD donors and LD were younger compared to DBD donors. Other baseline differences are shown in Table 1.

### Graft Survival

**FIGURE 2:**
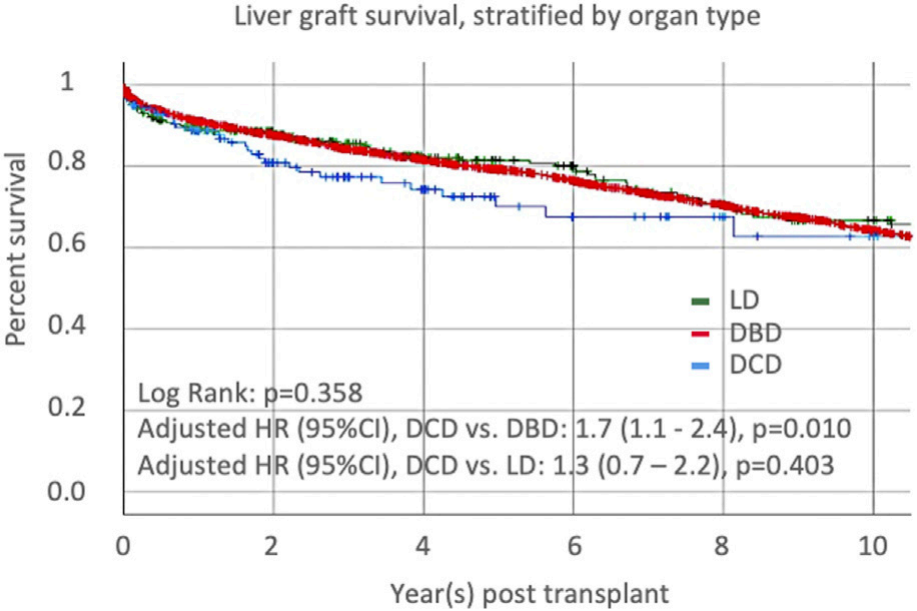
Liver graft survival, stratified by organ type: donation after cardiac death (DCD), donation after brainstem death (DBD), or living donor liver transplantation (LD).

Univariate Cox proportional-hazards regression identified several recipient and donor factors associated with graft loss (Table 2). After adjustment for variables with *p*-value<0.1 in the univariate model or key variable of interest (graft type), the multivariate Cox regression model identified older recipient age and Asian race, the presence of cholangiocarcinoma, era, the use of a DCD graft (compared to a DBD graft), a graft from a Native Hawaiian donor, older donor age, and increased CIT as independent risk factors for graft loss. CIT was not different between Native Hawaiian donors and non-Native Hawaiian donors, 9.7 h vs. 8.6 h, *p* = 0.132. Recipients receiving DCD grafts were 1.7 times more likely to lose their graft compared to DBD grafts, *p* = 0.010, and 1.3- times more compared LD grafts, *p* = 0.410. Protective factors against graft loss included Asian recipient ethnicity and recent transplantation era. We represented the distribution of groups within the different era (Supplementary Figure S2A) and confirmed the improvement of outcomes, overall and for DCD-LT, DBD-LT, and LD-LT independently (Supplementary Figures S2B,C). We confirmed that Graft survival at 1- and 5-year were 88.6% and 70.0% for DCD-LT, 92.6% and 79.9% for DBD-LT, and, 91.7% and 82.9% for LD-LT. Kaplan-Meier graft survival curves are shown in Figure 2.

**TABLE 2:** Estimated hazard ratios for liver graft survival using a uni-/multivariate Cox proportional hazard model.

Variables	Univariate analysis^a^			Multivariate analysis^b^		
	HR	95% CI	P-value	HR	95% CI	P-value
Recipient factors						
Age at transplant, years	1.0	1.0–1.0	0.029	1.0	1.0–1.0	0.002
Gender, male	1.0	0.9–1.2	0.323	NA	NA	NA
Pretransplant BMI, kg/m^2^	1.0	1.0–1.0	0.109	NA	NA	NA
Race/Ethnicity						
American Indian	1.3	0.8–2.2	0.368	NA	NA	NA
Asian	0.8	0.7–1.0	0.014	0.8	0.7–1.0	0.013
African American	1.2	0.9–1.5	0.126	NA	NA	NA
Native Hawaiian	0.5	0.2–1.2	0.123	NA	NA	NA
Hispanic	0.9	0.8–1.0	0.073	0.9	0.7–1.0	0.128
Multiracial	0.6	0.2–1.6	0.288	NA	NA	NA
Etiology						
Auto-immune	0.8	0.5–1.1	0.099	0.8	0.6–1.1	0.217
Amyloidosis	0.1	0.0 - NR	0.797	NA	NA	NA
Biliary atresia	1.8	0.4–7.2	0.412	NA	NA	NA
Cholangiocarcinoma	3.8	1.4–10.2	0.008	4.4	1.6–11.8	0.004
Cryptogenic	1.1	0.9–1.4	0.217	NA	NA	NA
EtOH	1.1	0.9–1.3	0.499	NA	NA	NA
HBV	0.9	0.7–1.1	0.272	NA	NA	NA
HCV	1.2	1.0–1.3	0.026	1.1	1.0–1.3	0.071
NASH	0.8	0.5–1.1	0.178	NA	NA	NA
PBC	1.0	0.8–1.3	0.926	NA	NA	NA
PSC	1.0	0.8–1.3	0.873	NA	NA	NA
Wilson	0.1	0.2–1.0	0.049	0.0	0.0 - NA	0.862
A1AT	0.4	0.1–1.5	0.173	NA	NA	NA
Other/unknown	0.9	0.8–1.1	0.253	NA	NA	NA
HCC	1.1	0.9–1.3	0.331	NA	NA	NA
MELD	1.0	1.0–1.0	0.037	1.0	1.0–1.0	0.945
Era						
1990–2000	NA	1 [Reference]	NA	NA	NA	NA
2001–2010	0.7	0.6–0.8	<0.001	0.7	0.6–0.8	<0.001
2011–2018	0.6	0.5–0.8	<0.001	0.6	0.5–0.7	<0.001
Donor factors						
Donor type						
DCD	NA	1 [Reference]	NA	NA	NA	NA
DBD	0.8	0.6–1.2	0.250	0.6	0.4–0.9	0.010
LD	0.7	0.5–1.1	0.153	0.8	0.5–1.4	0.403
Age, years	1.0	1.0–1.0	<0.001	1.0	1.0–1.0	<0.001
Gender, male	0.9	0.8–1.1	0.304	NA	NA	NA
BMI, kg/m^2^	1.0	1.0–1.0	0.737	NA	NA	NA
Race/Ethnicity						
American Indian	0.7	0.3–2.0	0.537	NA	NA	NA
Asian	1.0	0.8–1.2	0.794	NA	NA	NA
African American	1.2	0.9–1.4	0.185	NA	NA	NA
Hispanic	1.0	0.8–1.1	0.571	NA	NA	NA
Multiracial	0.6	0.3–1.4	0.217	NA	NA	NA
Native Hawaiian	1.6	1.0–2.7	0.073	2.2	1.3–3.6	0.004
Unknown	0.8	0.3–2.1	0.631	NA	NA	NA
White	1.0	0.9–1.1	0.991	NA	NA	NA
Cause of death						
Anoxia	0.8	0.8–0.9	0.010	0.9	0.7–1.1	0.332
Cerebrovascular	1.2	1.1–1.3	0.004	1.0	0.8–1.1	0.580
Head trauma	1.0	0.8–1.1	0.434	NA	NA	NA
CNS tumor	1.2	0.4–3.1	0.758	NA	NA	NA
Other	1.1	0.9–1.3	0.566	NA	NA	NA
Not reported	0.9	0.7–1.1	0.291	NA	NA	NA
Cold ischemic time, hours	1.0	1.0–1.0	0.015	1.0	1.0–1.0	0.026

DCD, Donation after cardiac death; DBD, donation after brainstem death; LD, living donor; LT, liver transplantation; BMI, body mass index, EtOH, ethanol use; HBV, hepatitis B virus; HCV, hepatitis C virus; NASH, nonalcoholic steatohepatitis, PBC, primary biliary cholangitis; PSC, primary sclerosing cholangitis; A1AT, alpha-1 antitrypsin; MELD, Model For End-Stage Liver Disease; CNS, central nervous system; BMI, body mass index; CI, confidence interval; HR, hazard ratio; NR, not reported (values superior to 10^6^); DCD, donation after cardiac death; DBD, donation after brainstem death (DBD); LD, living donor.

^a^ Univariate Cox proportional-hazards regression model.

^b^ Multivariate Cox regression model. Only those variables with *p* < 0.1 or of key clinical interest (graft type) in the univariate analysis were entered in the multivariate analysis.

Outcomes of donor after cardiac death liver transplant recipients compared to paired donation after brain death and living donor recipients.

Three groups of 138 LT recipients were constituted based on graft donation type (Table 3). Propensity matching allowed correction for most of the baseline variables between DCD and DBD donor/recipient characteristics. The etiology of liver disease, MELD score, HCC status, and era remained significantly different between groups. Out of 318 LD-LT recipients, 138 were randomly selected to be compared to DCDs. The sensitivity analysis included 265 LD-LT recipients. The baseline differences between the whole dataset and either the randomly matched or the propensity score-matched group remained unchanged. The differences in organ survival curves between the three donor types were mostly unchanged compared to the whole dataset (Supplementary Figure S1). Overall, eighteen LT recipients (4.3%, 18/414) had arterial complications (thrombosis and stenosis; no difference between groups). Eighteen patients (4.3%) were retransplanted, and there was no significant difference in retransplant rate between groups.

**TABLE 3:** Recipient and donor baseline characteristics of donation after cardiac death donation, and matched/paired control recipients receiving a graft after brainstem death and living donor.

Characteristics	DCD LT (*n* = 138)	DBD LT (*n* = 138)	LD LT (*n* = 138)	P-value^a^	P-value^b^
Recipient					
Age at tx. years	57.5 ± 9.0	57.8 ± 10.6	55.0 ± 11.2	0.797	0.041
(min–max)	(22–75)	(18–72)	(18–75)		
Gender (%)					
Male	103 (74.6)	98 (71.0)	67 (48.6)	0.499	<0.001
Female	35 (25.4)	40 (29.0)	71 (51.4)		
Pretransplant BMI. kg/m^2^	27.9 ± 5.9	27.4 ± 5.6	25.8 ± 4.3	0.478	0.001
Race/Ethnicity (%)					
White	75 (54.3)	64 (46.4)	78 (56.5)	0.053	0.583
African American	4 (2.9)	15 (10.9)	6 (4.3)		
Hispanic	38 (27.5)	29 (21.0)	41 (29.7)		
Asian	18 (13.0)	27 (19.6)	12 (8.7)		
Hawaii	1 (0.7)	2 (1.4)	1 (0.7)		
American Indian	2 (1.4)	1 (0.7)	0 (0.0)		
Etiology					
A1AT	1 (0.7)	1 (0.7)	2 (1.4)	<0.001	<0.001
Auto-immune	4 (2.9)	0 (0.0)	7 (5.1)		
Amyloidosis	1 (0.7)	0 (0.0)	0 (0.0)		
Biliary atresia	1 (0.7)	0 (0.0)	2 (1.4)		
Cholangiocarcinoma	2 (1.4)	2 (1.4)	0 (0.0)		
Cryptogenic	3 (2.2)	8 (5.8)	6 (4.3)		
EtOH	29 (21.0)	4 (2.9)	23 (16.7)		
HBV	12 (8.7)	18 (13.0)	11 (8.0)		
HCV	68 (49.3)	76 (55.1)	31 (22.5)		
NASH	9 (6.5)	5 (3.6)	19 (13.8)		
Other	0 (0.0)	3 (2.2)	12 (8.7)		
PBC	2 (1.4)	0 (0.0)	11 (8.0)		
PSC	3 (2.2)	17 (12.3)	14 (10.1)		
Wilson	3 (2.2)	4 (2.9)	0 (0.0)		
HCC					
Presence	40 (29.0)	60 (43.5)	27 (19.6)	0.012	0.068
Absence	98 (71.0)	78 (56.5)	111 (80.4)		
MELD	22.8 ± 11.1	34.2 ± 5.9	18.6 ± 7.5	<0.001	<0.001
Era					
1990–2000	0 (0.0)	19 (13.8)	0 (0.0)	<0.001	<0.001
2001–2010	26 (18.8)	31 (22.5)	1 (0.7)		
2011–2018	112 (81.2)	88 (63.8)	137 (99.3)		
Donor factors					
Age, years	31.7 ± 10.3	31.5 ± 13.5	35.7 ± 10.5	0.912	0.001
Gender (%)					
Male	92 (66.7)	87 (63.0)	64 (46.4)	0.528	0.001
Female	46 (33.3)	51 (37.0)	74 (53.6)		
BMI, kg/m^2^	25.5 ± 5.2	25.9 ± 7.1	25.6 ± 4.0	0.533	0.849
Race/Ethnicity (%)					
American Indian	0 (0.0)	2 (1.4)	0 (0.0)	0.293	0.157
Asian	4 (2.9)	8 (5.8)	12 (8.7)		
African American	11 (8.0)	11 (8.0)	5 (3.6)		
Hispanic	34 (24.6)	43 (31.2)	39 (28.3)		
Multiracial	4 (2.9)	2 (1.4)	4 (2.9)		
Hawaii	0 (0.0)	1 (0.7)	1 (0.7)		
White	85 (61.6)	71 (51.4)	77 (55.8)		
Cause of death					
Anoxia	73 (52.9)	40 (29.0)	NA	<0.001	NA
Cerebrovascular	17 (12.3)	35 (25.4)			
Head trauma	41 (29.7)	62 (44.9)			
Not reported	7 (5.1)	1 (0.7)			
Cold ischemic time, hours	7.7 ± 2.6	7.6 ± 3.5	2.1 ± 1.5	0.680	<0.001

Data are presented as mean ± standard deviation or n (%).

DCD, donation after cardiac death; DBD, donation after brainstem death; LD, living donor; LT, liver transplantation; BMI, body mass index, EtOH, ethanol use; HBV, hepatitis B virus; HCV, hepatitis C virus; NASH, nonalcoholic steatohepatitis; PBC, primary biliary cholangitis; PSC, primary sclerosing cholangitis; A1AT, alpha-1 antitrypsin; MELD, Model For End-Stage Liver Disease; CNS, central nervous system.

^a^ DCD versus DBD.

^b^ DCD versus LD. Student t-test for continuous variables, X^2^ test for binary or categorical variables (global *p*-value).

### Biliary Complications

**FIGURE 3:**
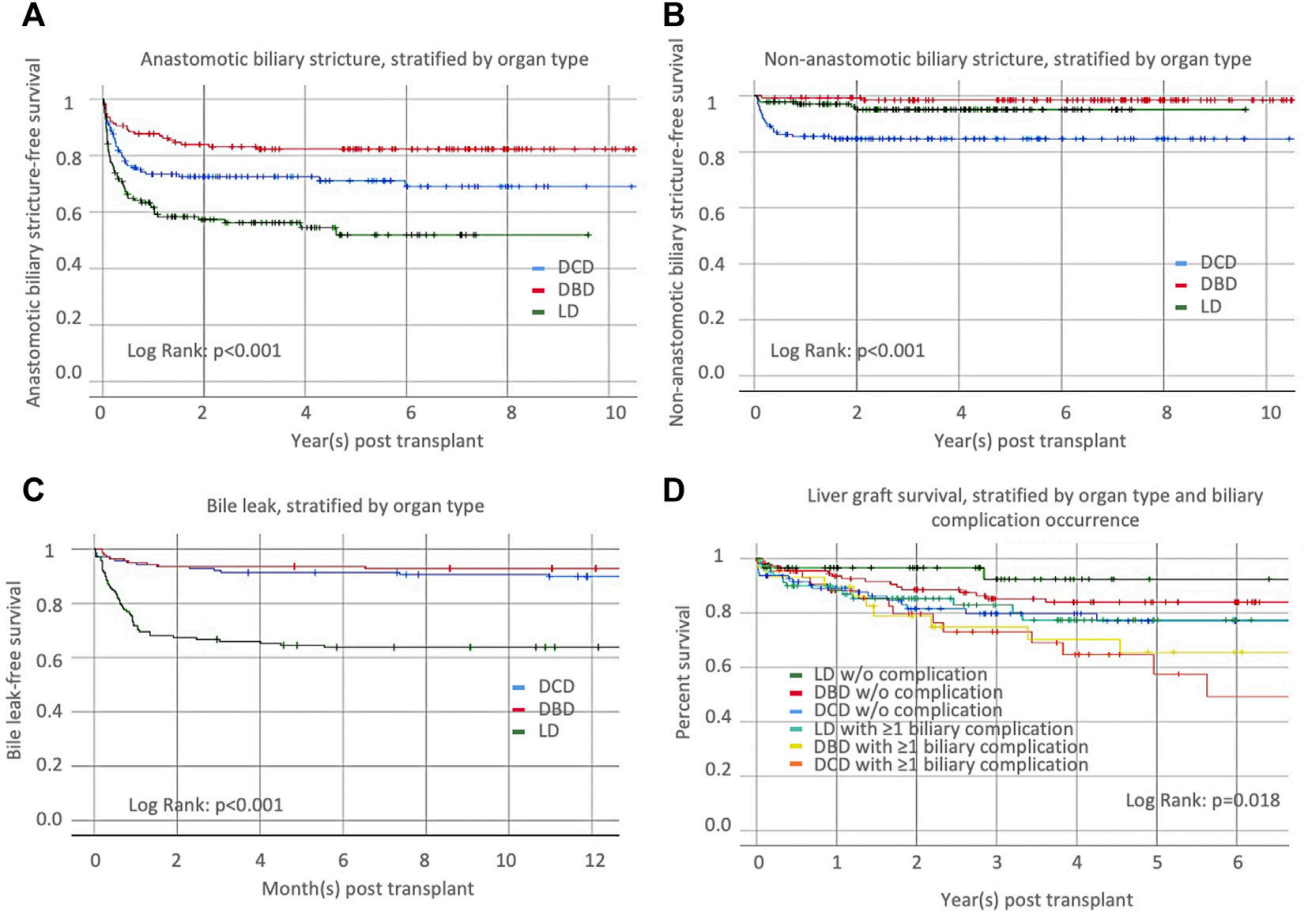
Occurrence of **(A)** anastomotic biliary stricture, **(B)** non-anastomotic biliary stricture, and **(C)** bile leaks, stratified by organ type [donation after cardiac death (DCD), donation after brainstem death (DBD), or living donor liver transplantation (LD)]. **(D)** Liver graft survival, stratified by organ type and biliary complication occurrence.

We studied the occurrence of anastomotic and non-anastomotic stricture and bile leak in the 414 adult recipients selected, as noted above. Anastomotic biliary strictures occurred in 28.3% of DCD recipients. Compared to DCD-LT, DBD recipients had fewer anastomotic strictures (18.1%), and LD recipients had more anastomotic strictures (43.5%) (Table 4; Figure 3A). Non-anastomotic biliary strictures developed in 15.2% of DCD recipients versus 1.4% of DBD recipients and 4.3% of LD recipients (Table 4; Figure 3B). NASs were observed much sooner after transplant in DCDs (median: 59 days) compared to DBDs (median: 409 days) or LDs (median: 172 days). Bile leak was observed in 10.1% of DCD recipients versus 7.2% of DBD recipients and 36.2% of LD recipients (Table 4; Figure 3C). Bile leaks usually occur between two to four weeks post-transplant. Patients who had leaks (regardless of the donor group) had more than a 4-time risk of developing AS and a three-time risk of developing NAS [HR (95%CI), 4.4 (3.1–6.4), *p* < 0.001 and HR (95%CI), 3.5 (1.7–7.4), *p* = 0.001), respectively]. Graft survival in the three groups (*n* = 414) was further stratified by organ type and biliary complication occurrence (none versus any) (Figure 3D). LD-LTs free of any biliary complications had the best graft survival, whereas DCD-LTs with ≥1 biliary complication (presence of a bile leak and/or AS and/or NAS) had the worst graft survival (global *p* = 0.018) (Figure 3D). Among the 21 DCD-LT patients with non-anastomotic strictures, six died (contraindication to retransplantation), two were retransplanted, three remain stent dependent, and notably, half (*n* = 10) are ultimately stent-free. LT recipients with NAS had worse graft survival compared to the NAS-free patients (*p* < 0.05). Patients with NAS had a median (IRQ) of 7 (5–10) ERCPs. We searched potential risk factors for any biliary complications in the matched/paired cohort (*n* = 414) (Table 5). After multivariate adjustment, the use of DBD grafts/donors with head trauma were found to be a protective factor against the occurrence of biliary complication(s). Higher donor BMI was associated with more biliary complications.

**TABLE 4:** Biliary complications in liver transplant recipients after receiving a liver from a cardiac death donor, a brainstem death donor, or living donor.

Characteristics	DCD LT (*n* = 138)	DBD LT (*n* = 138)	LD LT (*n* = 138)	Odds ratio P-value^a^	Odds ratio P-value^b^
Anastomotic biliary stricture	39 (28.3)	25 (18.1)	60 (43.5)	0.5 (0.3–0.9)	1.8 (1.2–2.6)
Time to stricture, d (min–max)	87.7 (0.0–2,191.5)	98.6 (0.0–5,113.5)	54.8 (0.0–1,826.3)	0.018	0.006
Non-anastomotic biliary stricture	21 (15.2)	2 (1.4)	6 (4.3)	0.1 (0.0–0.4)	0.3 (0.1–0.7)
Time to stricture, d (min–max)	59.0 (24.0–551.0)	409.0 (53.0–765.0)	172.0 (18.0–722.0)	0.001	0.005
Bile leak (%)	14 (10.1)	10 (7.2)	50 (36.2)	0.7 (0.3–1.6)	4.2 (2.3–7.7)
Time to bile leak, d (min–max)	27.5 (1.0–334.0)	16.5 (6.0–199.0)	17.5 (1.0–169.0)	0.402	<0.001

Data are presented as median (minimum–maximum) or n (%).

DCD, donation after cardiac death; DBD, donation after brainstem death; LD, living donor; LT, liver transplantation.

^a^ DBD versus DCD.

^b^ LD versus DCD. Student t-test for continuous variables, X^2^ test for binary variables.

**TABLE 5:** Estimated hazard ratios for biliary complication (any versus none) using a uni-/multivariate Cox proportional hazard model.

Variables	Univariate analysis^a^			Multivariate analysis^b^		
	HR	95% CI	P-value	HR	95% CI	P-value
Recipient factors						
Age at transplant, years	1.0	1.0–1.0	0.427	NA	NA	NA
Gender, male	0.9	0.6–1.2	0.398	NA	NA	NA
Pretransplant BMI, kg/m^2^	1.0	1.0–1.0	0.960	NA	NA	NA
Race/Ethnicity						
American Indian	1.5	0.4–5.9	0.602	NA	NA	NA
Asian	0.6	0.4–1.0	0.069	1.2	0.6–2.2	0.637
African American	1.0	0.5–1.9	0.944	NA	NA	NA
Native Hawaiian	0.6	0.1–4.5	0.639	NA	NA	NA
Hispanic	1.3	0.9–1.9	0.115	NA	NA	NA
Etiology						
Auto-immune	2.2	1.1–4.6	0.026	1.3	0.6–2.9	0.437
Amyloidosis	0.1	0.0–NA	0.650	NA	NA	NA
Biliary atresia	1.1	0.2–7.8	0.934	NA	NA	NA
Cholangiocarcinoma	1.5	0.4–6.0	0.581	NA	NA	NA
Cryptogenic	1.1	0.5–2.4	0.781	NA	NA	NA
EtOH	1.2	0.8–1.8	0.486	NA	NA	NA
HBV	0.4	0.2–0.9	0.020	0.4	0.2–1.1	0.067
HCV	0.7	0.5–1.0	0.079	0.9	0.6–1.4	0.768
NASH	1.1	0.6–1.9	0.804	NA	NA	NA
PBC	2.4	1.1–5.0	0.027	1.4	0.6–3.2	0.396
PSC	1.1	0.6–1.9	0.713	NA	NA	NA
Wilson	0.7	0.2–2.8	0.623	NA	NA	NA
A1AT	2.8	0.9–8.7	0.082	2.7	0.8–8.8	0.108
Other/unknown	2.1	1.1–3.9	0.027	1.3	0.6–2.6	0.477
HCC	0.7	0.5–1.0	0.033	0.9	0.6–1.4	0.693
MELD	1.0	1.0–1.0	<0.001	1.0	1.0–1.0	0.785
Era						
1990–2000	NA	1 [Reference]	NA	NA	NA	NA
2001–2010	2.7	0.8–9.7	0.115	1.3	0.3–5.0	0.737
2011–2018	4.0	1.2–13.2	0.024	1.0	0.3–3.8	0.993
Donor factors						
Donor type						
DCD	NA	1 [Reference]	NA	NA	NA	NA
DBD	0.6	0.4–0.9	0.022	0.6	0.3–1.0	0.049
LD	2.4	1.7–3.5	<0.001	1.7	0.9–3.1	0.094
Age, years	1.0	1.0–1.0	0.002	1.0	1.0–1.0	0.260
Gender, male	0.7	0.5–1.0	0.030	0.9	0.7–1.3	0.729
BMI, kg/m^2^	1.0	1.0–1.1	0.028	1.0	1.0–1.1	0.029
Race/Ethnicity						
American Indian	0.0	0.0–388.7	0.511	NA	NA	NA
Asian	1.1	0.6–2.1	0.731	NA	NA	NA
African American	1.3	0.7–2.3	0.437	NA	NA	NA
Hispanic	1.0	0.7–1.4	0.994	NA	NA	NA
Multiracial	0.5	0.1–1.9	0.299	NA	NA	NA
Native Hawaiian	1.5	0.2–11.1	0.664	NA	NA	NA
White	1.0	0.7–1.4	0.916	NA	NA	NA
Cause of death						
Anoxia	0.6	0.4–0.8	0.005	0.6	0.3–1.1	0.094
Cerebrovascular	1.1	0.7–1.7	0.659	NA	NA	NA
Head trauma	0.4	0.2–0.6	<0.001	0.5	0.3–0.9	0.028
Other	0.6	0.1–2.4	0.462	NA	NA	NA
Cold ischemic time, hours	0.9	0.9–0.9	0.000	1.0	1.0–1.1	0.249

DCD, donation after cardiac death; DBD, donation after brainstem death; LD, living donor; LT, liver transplantation; BMI, body mass index, EtOH, ethanol use; HBV, hepatitis B virus; HCV, hepatitis C virus; NASH, nonalcoholic steatohepatitis; PBC, primary biliary cholangitis; PSC, primary sclerosing cholangitis; A1AT, alpha-1 antitrypsin; MELD, Model For End-Stage Liver Disease; CNS, central nervous system; BMI, body mass index; CI, confidence interval; HR, hazard ratio; NR, not reported (values superior to 105); DCD, donation after cardiac death; DBD, donation after brainstem death (DBD); LD, living donor.

^a^ Univariate Cox proportional-hazards regression model.

^b^ Multivariate Cox regression model. Only those variables with *p* < 0.1 in the univariate analysis were entered in the multivariate analysis.

## Discussion

Liver transplantation using DCD or LD donors is limited to a minority of centers because of the higher rates of ischemic cholangiopathy (DCD-LTs) or biliary complications (DCD and LD-LTs) compared with grafts from DBD donors [7, 11, 14, 23, 27]. Nevertheless, DCD- and LD-LTs often represent the only life-saving option for specific liver recipient candidates in a MELD-based allocation system. For these patients, the benefits of receiving a DCD or LD graft have the potential to increase survival and quality of life compared to staying on the transplant waiting list. Over the last few years, refinement in donor and recipient selection has allowed a significant improvement in outcomes for DCD-LT [11, 12, 14]. However, for many patients, waiting for a DBD, involving an LD, or taking a DCD offer remains a common dilemma. We thus sought to analyze and compare the outcomes and biliary complications of DCDs to DBDs and LDs in a single-center LT recipient population.

We first observed and confirmed the known increased risk of graft loss (HR of 1.7) in recipients receiving DCD livers compared to those receiving DBD grafts, which matches with previously reported risk [28, 29], although the most recent cohort studies suggest this HR was further lowered [30, 31]. The comparison between DCD-LTs and LD-LTs was not significant, possibly due to the lower number of patients in the latter group. Nevertheless, graft survival curves showed that all three categories converged over time, suggesting that DCD is an acceptable alternative when no other organ is available. The graft survival rates for the three categories matches those observed and reported by the Toronto group in a similar analysis [32]. We identified other important predictors of graft loss and patient death in our multivariate analysis, including donor and recipient age, transplantation era, presence of cholangiocarcinoma, and cold ischemia time, considering previously described risk factors [28]. We also highlighted a detrimental effect of donor Hawaiian ethnicity and a protective effect of recipient Asian ethnicity.

The nature and frequency of biliary complications are what differentiate most long-term outcomes in DCD-LT versus DBD-LT or LD-LT. A focused analysis led us to study biliary complications in 414 recipients, including one-third of each donor type. Non-anastomotic biliary stricture developed in 15.2% of DCD recipients, which aligns with what is reported in the literature [7, 8, 11]. This complication was exceptional in DBD-LT or LD-LT in the absence of an arterial supply problem. There was a slight increase in anastomotic biliary strictures and bile leaks in DCD-LT recipients compared to DBD-LT recipients, but the increase was not prohibitive. Living donor recipients had a higher number and completely different pattern of biliary complications compared to the two other groups, as previously reported [32]. They were much more affected with anastomotic biliary strictures (43.5%) and bile leaks (36.2%) compared to DCD-/DBD-LT recipients. It is worth noting that recipients with bile leaks are the group that typically get strictures. Taken together, the type of transplant and the presence of biliary complications had an impact on organ survival. The best 1- and 5-year graft survival were achieved in LD recipients without biliary complication and the worst in DCD recipients with any type of biliary complication. This was further confirmed in a multivariate analysis where DBD grafts/donors with head trauma were the only protective factors against the occurrence of biliary complication(s). It is unclear why higher donor BMI was associated with more biliary complications. This could be a marker of graft quality (steatosis) which could have an impact on the magnitude of ischemia-reperfusion injury and biliary microcirculation damage. However, it is important to note that the magnitude of this association was limited.

The development of NAS negatively affected graft survival; however, 50% of the patients with NAS ultimately kept their graft and remained stent free in the long term.

Overall, given the reported 1- and 5-year graft survival rates and biliary complication rates, it seems that both DCD-LT and LD-LT are viable options when DBD grafts are limited or unavailable. Successful LD selection is well codified, and biliary complication rates vary between different centers [23]. Similarly, DCD donor and recipient selection criteria are center-dependent and may affect survival outcomes and the rate of biliary complications [6–9]. Large discrepancies exist in DCD utilization, the most striking one being the difference between the United States and the United Kingdom: DCD LT currently accounts for about 8% of all deceased donor LTs in the US versus 19% in the United Kingdom [9]. In our center, general DCD selection criteria included donor age younger than 60, an estimated CIT lower than 8 h, dWIT<30 min, and a recipient with a MELD score lower than the average. Several DCD scores [11, 12, 14], including ours [13], have been published to further standardize practices and ensure the best outcomes; however, local constraints (travel distance, local MELD, etc.) and practices can make these scores hard to follow in a global and protocoled manner.

Our study has limitations. We report on a retrospective cohort; thus, information bias and selection bias cannot be totally avoided. It is noteworthy that the number of missing data was low, therefore limiting information bias. Another point is that our study extends over a large period (especially for DBDs and LDs); therefore, we cannot totally exclude bias related to the evolution of surgical technique, donor/recipient selection practices, and recipient management policies. To account for this, we used “era” as an independent study variable in our multivariate analysis. Interestingly, era was significantly associated with graft survival but not with biliary complications. Moreover, the low number of DCDs and LDs and the fact that the evolution of techniques is a continuum prompted us to consider a larger study period. However, to limit its impact, we restricted the matched/paired analysis to the 2003–2019 period. Another limitation is that our conclusions are based on a single-center data analysis and should be confirmed in a multicenter cohort. From a broader perspective, it is also worth noting that the increasing use of machine perfusion devices for DCDs may change the rate and nature of complications in the future [10, 33–36]. Normothermic regional perfusion [37–39] and hypothermic oxygenated perfusion [40] might indeed have an impact on the prevention of biliary complications. To date, it remains to be demonstrated whether the *ex situ* perfusion technologies will lead to a significant risk modification that is proportional to the costs and logistic difficulties of their use and/or if this risk modification can be achieved through better organ selection. Nevertheless, the choice of proceeding with an LD versus waiting for a DCD or a DBD graft to become available will remain a point of discussion globally and at the individual patient level.

In conclusion, we exposed the differential incidence and effect of biliary complications on the outcomes after liver transplantation using brain-dead donors, donors after circulatory death, and living donors. We demonstrated that LD-LT achieved the best 1-and 5-year graft survival, and DCD-LT achieved excellent graft survival in the absence of biliary complications. DCD-LT is expected to become an equivalent alternative to DBD- and LD-LT given the further reduction of ischemia-reperfusion injury and biliary microcirculation damage offered by machine and regional perfusion systems.

## Data Availability

The raw data supporting the conclusion of this article will be made available by the authors, without undue reservation.

## Supplementary Material

Supplementary Figure S1 Liver graft survival, stratified by organ type: donation after cardiac death (DCD), donation after brainstem death (DBD), or living donor liver transplantation (LD) in the matched/paired cohort.

Supplementary Figure S2
**(A)** Relative numbers of liver transplant (%) in the unmatched and matched cohorts. **(B)** Liver graft survival stratified by era (all liver transplant types). **(C)** Liver graft survival, stratified by organ type: donation after cardiac death (DCD), donation after brainstem death (DBD), or living donor liver transplantation (LD), and within the different era.
