# Supplementary figures and images for: Comparison of Biliary Complications Rates After Brain Death, Donation After Circulatory Death, and Living-Donor Liver Transplantation: A Single-Center Cohort Study
Source: Transpl Int. 2022 Dec 9;35:10855. doi: 10.3389/ti.2022.10855 (PMC9780276; doi:10.3389/ti.2022.10855)

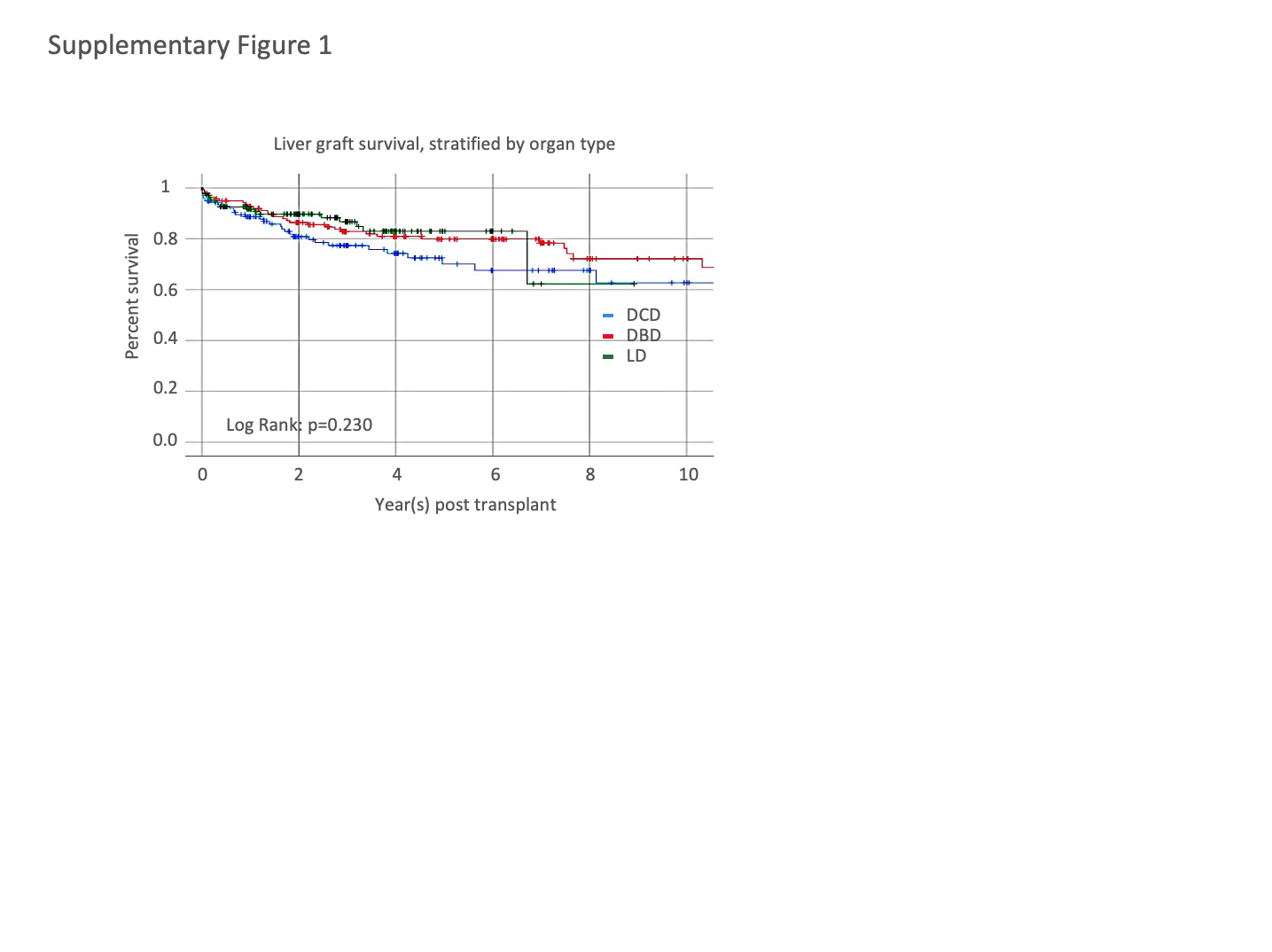

Supplement: Supplementary file 1 [file Image1.jpeg]

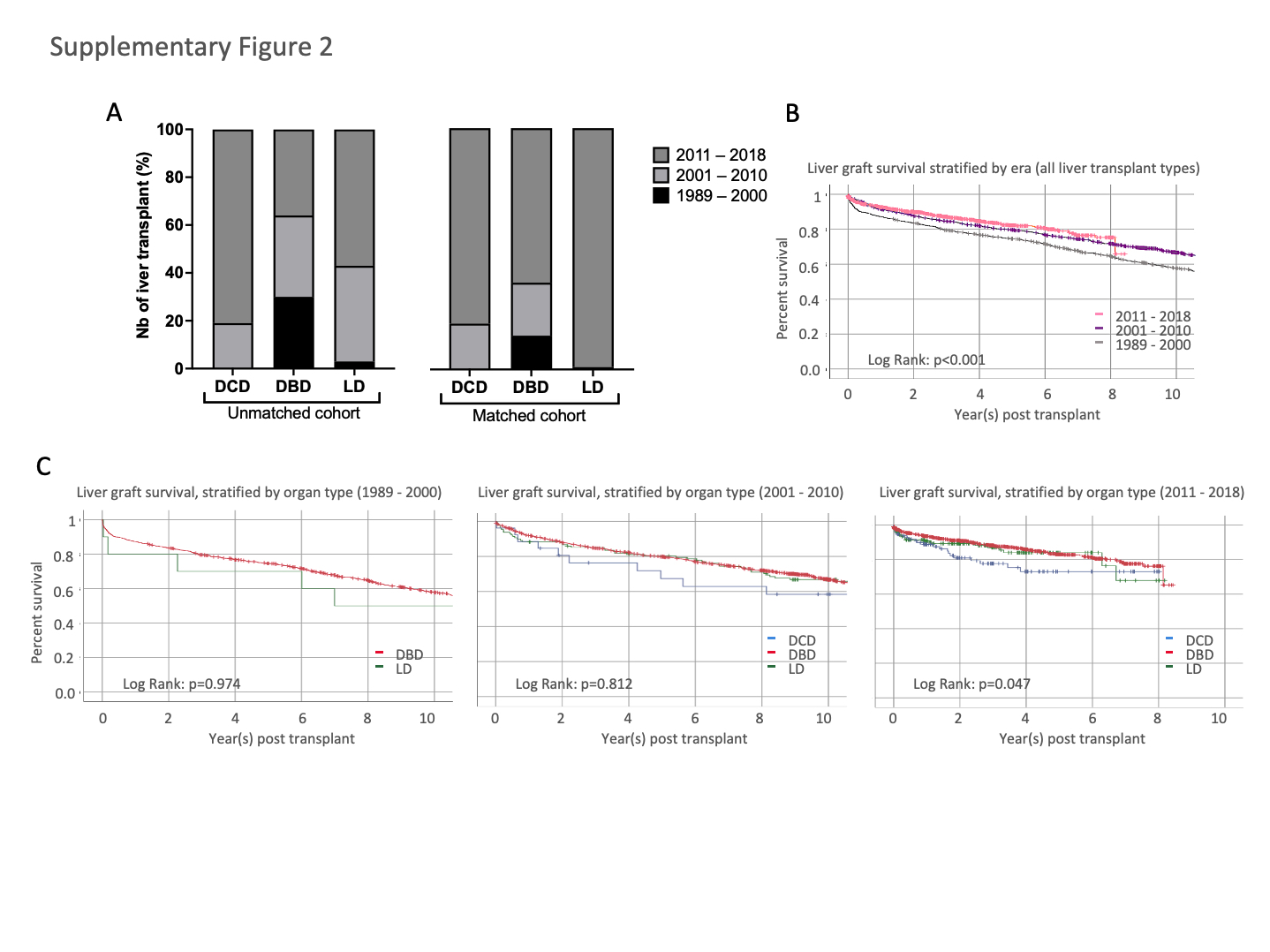

Supplement: Supplementary file 2 [file Image2.jpeg]
